# Identification of serum biomarkers associated with microvascular functions in a long-term high-fat diet-induced obesity rat model

**DOI:** 10.1590/1414-431X2025e14811

**Published:** 2025-08-29

**Authors:** S. Elmas, E.G. Cekic, P. Cenik, F. Sirinyildiz, O. Elmas, O. Elmas, G. Cesur

**Affiliations:** 1 Department of Biology, Faculty of Science, Mugla Sitki Kocman University, Mugla, Turkey; 2 Department of Pharmacology, Faculty of Medicine, Mugla Sitki Kocman University, Mugla, Turkey; 3 Department of Physiology, Faculty of Medicine, Adnan Menderes University, Aydin, Turkey; 4 Biochemistry Laboratory, Korkuteli State Hospital, Antalya, Turkey; 5 Department of Physiology, Faculty of Medicine, Suleyman Demirel University, Isparta, Turkey

**Keywords:** High fat diet, Microvascular functions, Leptin, VEGF, MPO, CRP

## Abstract

Serum biomarkers are crucial for identifying complications of obesity. This study evaluated serum levels of leptin, vascular endothelial growth factor (VEGF), myeloperoxidase (MPO), C-reactive protein (CRP), oxidized low-density lipoprotein (OXLDL), low-density lipoprotein (LDL), and other biochemical parameters in a rat model of high-fat diet (HFD)-induced obesity to investigate potential relationships between these biomarkers and microvascular function. Rats in the HFD group were fed a high-fat diet for 23 weeks, whereas control rats received a standard diet. Microvascular function was assessed using the post-occlusive reactive hyperemia (PORH) test; PORH responses were measured in the right forelimbs using laser Doppler flowmetry. Serum samples were then collected to measure the aforementioned biomarkers. Results showed decreased PORH responses in the HFD group, indicating impaired microvascular function. Serum levels of leptin, MPO, CRP, LDL, and OXLDL were significantly higher in the HFD group. Strong correlations were observed between microvascular dysfunction and LDL, OXLDL, MPO, and CRP. No significant changes were found in VEGF or HDL levels. These findings suggest that increased LDL oxidation to OXLDL in obesity contributes to vascular impairment, likely due to increased oxidative stress and inflammation mediated by elevated MPO and CRP. Further research focusing on the roles of LDL, OXLDL, MPO, and CRP may provide deeper insights into the mechanisms underlying microvascular dysfunction in obesity.

## Introduction

According to the latest data from the World Health Organization (WHO), approximately 39% of the world's population aged 18 and older is overweight and 13% is obese. Overweight and obesity are responsible for 44% of the diabetes burden, 23% of the ischemic heart disease burden, and 7 to 41% of the burden of certain cancers (1). Every year, at least 2.8 million adults die as a result of complications from excess weight or obesity (2). The most common cause of mortality and morbidity in these patients is coronary artery disease (CAD), which is caused by increased atherosclerosis (2).

Post-occlusive reactive hyperemia (PORH) is a transient increase in organ blood flow that occurs after a brief ischemic period and is used to assess endothelial function, which reflects microvascular health. Laser Doppler flowmetry, a widely used method in the assessment of PORH, provides accurate and reliable results in the evaluation of microvascular functions by measuring blood flow in capillaries non-invasively. Obese people have reduced PORH responses (3). Hyperglycemia, dyslipidemia, inflammation, and oxidative stress have been linked to impaired vasodilator control mechanisms in obese people (4). Although the mechanism is not fully understood, the impaired PORH in peripheral circulation is recognized as a predictor of CAD (5).

Leptin plays critical roles in obesity. Leptin is primarily produced by fat cells and functions to signal to the central nervous system that the existing energy stores are adequate, thereby limiting food intake (6). Blood leptin levels are directly correlated with body fat mass, and obese individuals produce approximately twice as much leptin as healthy individuals (7). There are some conflicts in the literature regarding the link between serum leptin levels and microvascular functions: leptin is a vasodilator that directly activates the nitric oxide (NO) pathway (8). The activation of the NO pathway implies a strong reactive hyperemia response. However, despite high leptin levels, obese people have low PORH responses (9). There are also various studies that focus on leptin administration and PORH responses; while some show that leptin improves PORH, others show no change (10,11).

Vascular endothelial growth factors (VEGF) are essential signaling proteins involved in both vasculogenesis (*de novo* formation of the embryonic circulatory system) and angiogenesis (the proliferation of pre-existing blood vessels) (12). Elevated levels of VEGF are observed in obese individuals (13). There are a limited number of animal model studies investigating VEGF levels in obesity. In one such study, obese rats fed a high-fat diet (HFD) demonstrated significantly elevated VEGF levels (14). Additionally, a study in mice genetically modified to suppress VEGF expression in adipose tissue demonstrated that these mice were resistant to weight gain despite being fed an HFD (15). These studies suggest a link between obesity and VEGF upregulation and VEGF's potential role in obesity-related vascular changes.

Some studies indicate that the leptin hormone can increase VEGF synthesis. It has been reported that leptin, through VEGF, enhances angiogenesis in the development of certain tumors and tissue repair (16,17). Furthermore, leptin directly upregulates and transactivates VEGF receptors, as shown in cell culture studies and experiments involving human cells and rat corneas (18,19). It is plausible that, similar to its effects in tumors, leptin may increase VEGF production in obesity. If leptin enhances VEGF effectiveness, this could theoretically improve microvascular function. However, despite elevated leptin and VEGF levels in obese patients, PORH responses remain paradoxically low, suggesting a discrepancy between these molecular changes and microvascular outcomes.

Plasma myeloperoxidase (MPO) and C-reactive protein (CRP) levels, similar to PORH responses, are considered predictors of cardiovascular incidents (20). MPO is produced in the azurophilic granules of neutrophils and monocytes. MPO plays a significant role in the innate immune system by facilitating the formation of microbicidal reactive oxidants. Elevated MPO levels in circulation are associated with inflammation and increased oxidative stress (20). On the other hand, CRP is primarily synthesized in hepatocytes of the liver, as well as in smooth muscle cells, macrophages, endothelial cells, lymphocytes, and adipocytes. CRP plays crucial roles in host responses to infection, including the complement pathway, apoptosis, phagocytosis, NO release, and cytokine production (21). Although its mechanism has not been fully elucidated, it is believed that the increase in lipid content in adipose tissue leads to an increase in proinflammatory mediators produced by resident immune cells in adipose tissue, and that these mediators contribute to the increased production of MPO and CRP (22). It has been reported that leptin increases proinflammatory cytokines, leading to vascular inflammation, oxidative stress, and vascular smooth muscle hypertrophy (23). It is possible that these effects may also be due to MPO, CRP, or other inflammatory or oxidative mediators associated with obesity.

MPO and CRP may contribute to impaired microvascular function in obesity. Studies on human subjects have reported correlations between MPO and decreased PORH, and in hyperlipidemic individuals, elevated serum CRP levels have been associated with decreased PORH (24,25). Similarly, in obese rat models, increased MPO activity has been linked to endothelial dysfunction and microvascular disorders, while MPO inhibition has improved endothelial function and reduced inflammation (26). In mouse models, CRP has been shown to reduce the synthesis of endothelial-derived vasodilator substances and induce microvascular dysfunction by impairing endothelial progenitor cell function (27). However, the literature also contains conflicting findings. Evidence suggests that immune cells co-secrete MPO and VEGF (28). Furthermore, multiple cell culture studies have demonstrated that CRP increases VEGF expression (29). Conversely, some studies indicate that CRP reduces VEGF receptors or has no significant role in microvascular dysfunction (30). The simultaneous production of VEGF, which increases PORH, and MPO, which decreases PORH, along with the observation that CRP both increases VEGF expression and reduces the synthesis of vasodilator substances, presents a seemingly paradoxical situation.

The existing literature to date paints a complex and often conflicting picture of the interplay between leptin, VEGF, MPO, CRP, and microvascular function in obesity. While individual studies have provided valuable insights, a comprehensive assessment of key biomarkers in obesity within a single cohort would resolve these contradictions. Therefore, the objective of this study was to utilize a rat model of obesity to systematically evaluate the serum levels of leptin, VEGF, MPO, and CRP, and their association with microvascular function, aiming to address the knowledge gap in this field and better understand the mechanisms contributing to microvascular dysfunction in obesity.

## Material and Methods

### Animals

The experiment was approved by Adnan Menderes University's Local Animal Ethics Committee (approval number: 64583101/2014/082). Twenty-three male Wistar Albino rats, weighing an average of 272±26.8 g at nine weeks of age, were included in the study. The rats were obtained from the Laboratory Animals Unit of the Adnan Menderes University Veterinary Faculty. They were divided into two groups: the control group (n=9) and the HFD group (n=14). The rats were housed in polycarbonate cages that maintained a humidity level of 40-60% and an ideal temperature of 22°C, with a 12-h light/dark cycle.

### Feeding of the animals and the obesity model

For the HFD group of rats, a high-calorie fatty feed was prepared (31). For this purpose, the standard rat feed was first ground into flour. To prepare 1 kg of feed, 600 g of ground standard feed was mixed with 400 g of animal tallow. The mixture was then formed into pellets. In this way, feed containing approximately 40% fat was prepared. The feeds were prepared every two days and kept in the refrigerator to avoid spoilage. The same procedures were applied to the feeds for the control group, except without the addition of fat. The energy and macronutrient contents of the feeds prepared for the control and HFD groups are given in Table 1. For 23 weeks, the control group rats were fed with fat-free standard rat feed, while the HFD group rats were fed with high-calorie feed. Both groups of rats had unrestricted access to food and drinking water from the tap.

**Table 1:** Approximate energy and macronutrient contents of the diets prepared for the control group and the high-fat diet (HFD) group.

Content	Control group diet		HFD group diet	
	g %	Cal %	g %	Cal %
Carbohydrate	54.1	69.8	32.5	25.5
Protein	20.0	23.0	13.3	9.26
Lipid	2.7	7.2	40.3	65.2
Total energy (Cal/g)	3.49		5.74	

The energy and macronutrient contents of the diets were obtained from the manufacturers or nutrition labels on the packages.

### Anthropometric measurements

The weight and length of the rats were measured, and their body mass index (BMI) and Lee index were calculated, both before and after the experiment (32). Body length was defined as the distance between the tip of the nose and the base of the tail. The weights were measured on a scale (Fakir, Scala, Cavory Industrial Co., China) and recorded in grams. BMI was calculated as body weight (g) / body length^2^ (cm^2^), and the Lee index was calculated as the cube root of body weight (g^1/3^) / body length (cm).

### PORH analysis

The hyperemic responses were examined using a non-invasive technique known as PORH-Laser Doppler Flowmetry (LDF) (33). The Perimed Periflux 5010 system (Perimed, Sweden) and a small flat skin probe (Probe 407) were used for LDF measurements. The Doppler flow recording was obtained and analyzed with Perisoft for Windows 2.55 (Perimed). The probe applies a laser light to the skin, and the reflected laser lights create a Doppler signal depending on the concentration and velocity of red blood cells. This signal is converted to perfusion units (PU) by the software. The calibration protocol was implemented with calibration kits providing standard values after the device was turned on and warmed up for 20 min.

**Figure 1:**
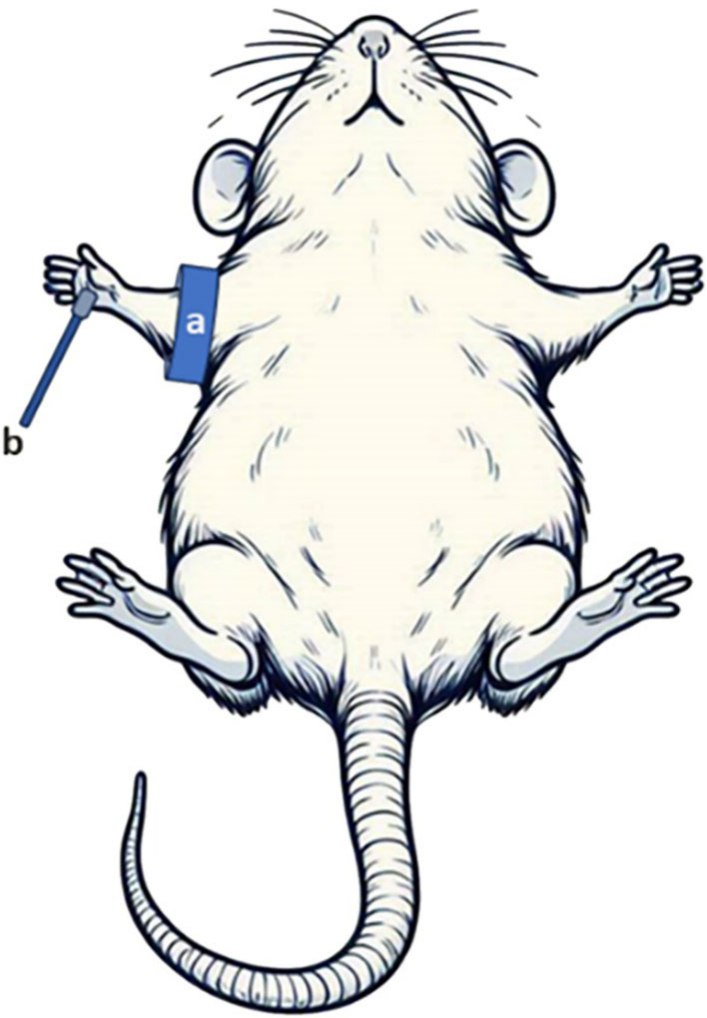
Probe and cuff placement for laser Doppler flow measurement. **a**, An inflatable pneumatic cuff connected to a sphygmomanometer was attached to the right upper front leg. **b**, The hairless midpoint of the right front paw's plantar surface of the rat was used to attach a Doppler flow probe.

LDF measurements were performed on rats under anesthesia. The Doppler probe was affixed to the hairless midpoint of the plantar surface (sole) of the rat's right front paw using adhesive tape, and a cuff was secured to the rat's right front upper leg, as illustrated in Figure 1.

**Figure 2:**
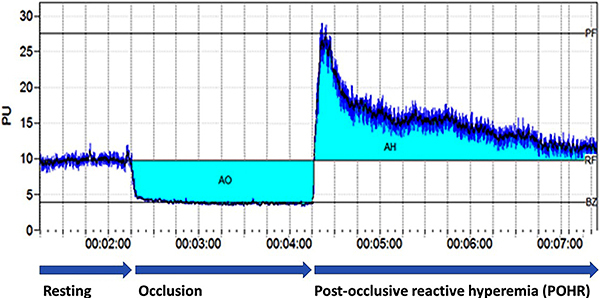
A sample of the laser Doppler flow recording obtained using Perisoft software. The figure depicts the parameters of post-occlusive reactive hyperemia. PF: peak flow; RF: resting flow; BZ: biological zero; AO: area of occlusion; AH: area of hyperemia.

Figure 2 shows a sample recording obtained with the software. First, the resting flow (RF) was recorded. Then, the cuff was rapidly inflated using a sphygmomanometer up to a pressure of 180 mmHg, and the brachial artery was temporarily occluded for 2 min. The PU value obtained during occlusion is referred to as the biological zero (BZ). At the 2nd minute, the cuff pressure was rapidly reduced, allowing for the restoration of blood flow to the right foot and the reperfusion of the right paw. Due to post-occlusive hyperemia, the peak flow (PF) value rises above the RF value. LDF measurement was continued until the perfusion returned to the resting level. In the study, RF, BZ, PF, PF-RF, the area of occlusion (AO), the area of hyperemia (AH), and the AO/AH ratio were used to evaluate PORH. AO represents the area below the RF line from the beginning of the occlusion to its end, while AH represents the area above the RF line from the end of the occlusion until the flow returns to the RF level.

### Laboratory assays

At the end of the experiment, the rats were anesthetized with an intraperitoneal injection of 10% ketamine HCl (90 mg/kg) and 2% xylazine HCl (10 mg/kg) (34). By using an intracardiac puncture, blood samples were collected into gel serum tubes. The sera were then transferred to Eppendorf tubes and the tubes were centrifuged for five minutes at room temperature at 1200 *g*. The samples were kept at -80°C in a deep freezer until the measurement day.

The Rat Leptin ELISA Kit (Sigma-Aldrich, USA, Catalog #: RAB0335) was used for the measurement of leptin levels, the Rat MPO ELISA kit (MyBioSource, USA, Catalog #: MBS704859) was used for the measurement of MPO levels, the Rat VEGF ELISA Kit (Abcam Inc., UK, Catalog #: ab100786) was used for the measurement of VEGF levels, the Rat LDL (low density lipoprotein) ELISA Kit (Elabscience, China, Catalog #: E-EL-R0579) was used for the measurement of LDL levels, and the Rat Oxidized Low Density Lipoprotein (OxLDL) ELISA Kit (MyBioSource Inc., Catalog #: MBS262297) was used for the measurement of OxLDL levels. ELISA measurements were performed according to the instructions provided with each kit using a Spectramax-i3 microplate reader (Molecular Devices, USA).

Serum CRP, HDL, total cholesterol (TCHOL), triglycerides (TG), glucose, and HbA1c were measured on an automated clinical chemistry analyzer (Erba Mannheim XL-640, Transasia Bio-Medicals LTD., India). CRP (Catalog #: XSYS0084) and HbA1c (Catalog #: XSYS0054) were measured using the immunoturbidimetric method, while HDL (Catalog #: XSYS0043), TCHOL (Catalog #: XSYS0009), TG (Catalog #: XSYS0041), and glucose (Catalog #: XSYS0012) were measured photometrically. All kits used in the device were obtained from the device manufacturer. The device and kit instructions were followed during the measurements.

### Statistical analysis

Statistical power analysis was conducted using GraphPad StatMate software (GraphPad Software Inc., USA) to determine the necessary sample size for detecting significant differences between groups. The power analysis was performed using parameters of a 5% significance level (α=0.05), an effect size (Cohen's d) of 1.0, and 80% statistical power (1-β=0.8), consistent with the literature review detailed in the Introduction and the findings of similar studies. Based on the power analysis, the minimum required sample size for each group was calculated as 8. However, considering the long duration of the experiment and potential animal losses, the sample size for each group was increased to 10. Additionally, since there was limited data in the literature for some parameters in the HFD group, the sample size for the HFD group was increased by 50%, as permitted by the ethics committee, to enhance the reliability of the reference values. Consequently, the control group was set at 10, and the HFD group was set at 15, as outlined above.

All statistical analyses of the collected data were performed using GraphPad Prism 6.0 software (GraphPad Software Inc.). The D'Agostino and Pearson's omnibus normality tests, as well as the Shapiro-Wilk normality test, were used to determine whether data from each group fell within a normal distribution. Subsequently, the Mann-Whitney U test was applied to examine any potential statistical disparities between the groups. Bonferroni correction was applied in the analysis of differences between groups. We selected the significance levels based on the conventional standards as follows: P<0.05 was considered statistically significant, P<0.01 was considered highly significant, and P<0.001 was considered extremely significant. The relationships between group parameters were assessed through Spearman correlation analysis. Relationships were classified as very strong (r≥0.8), strong (0.8>r≥0.6), moderate (0.6>r≥0.4), weak (0.4>r≥0.2), or negligible (r<0.2).

## Results

### Anthropometric measurements

Table 2 shows the weights, lengths, BMIs, and Lee indices of the rats in the HFD and control groups on the first day and at the end of the 23rd week. On the first day of measurements, there were no significant differences in body weight, length, BMI, or Lee index between the two groups. According to data obtained at the end of the 23rd week, the obese group had significantly higher body weight, and BMI compared to the control group. This outcome shows that the obesity model we used was effective. Rats in the HFD group had a higher average length and Lee index at the end of the 23rd week than rats in the control group, but the length differences were not statistically significant, while the Lee index differences were marginally significant.

**Table 2:** Anthropometric measurements of the rats in the control and high-fat diet (HFD) groups on the first day and at the end of the 23rd week.

	Body weight (g)		Length (cm)		Body mass index (g/cm^2^)		Lee Index (gr^1/3^/cm)	
	First day	After 23rd week	First day	After 23rd week	First day	After 23rd week	First day	After 23rd week
Control (n=9)	266.0±30.3	437.0±44.6	21.1±1.10	25.4±1.63	0.605±0.100	0.684±0.091	0.306±0.021	0.300±0.018
HFD (n=14)	277.0±21.0	520.0±43.8	20.7±1.86	28.8±1.82	0.665±0.136	0.789±0.101	0.318±0.031	0.313±0.019
P value	0.50	<0.001***	0.78	0.84	0.29	0.007**	0.32	0.054˟

Data are reported as means±SEM. **P<0.01, ***P<0.001, ˟marginally significant (Mann-Whitney U test with Bonferroni correction).

### PORH responses

Table 3 shows the RF, BZ, PF, AO, and AH values obtained from the PORH test conducted at the end of the 23rd week for both groups, along with the derived values of PF-RF and AH/AO. There were no differences between the two groups in terms of RF, BZ, and AO values. In the HFD group, PORH response indicators PF, PF-RF, AH, and AH/AO values were statistically decreased compared to the control group. This result indicated that the high-fat diet reduced PORH responses.

**Table 3:** Results of the post-occlusive reactive hyperemic response measured with laser Doppler flowmetry in the high-fat diet (HFD) and control groups at the end of the 23rd week.

	RF (PU)	BZ (PU)	PF (PU)	PF-RF (PU)	AO (PU·s)	AH (PU·s)	AH/AO
Control (n=9)	7.54±0.519	4.46±0.519	29.9±1.82	22.3±1.97	506±51.0	885±71.0	1.77±0.235
HFD (n=14)	7.31±0.630	4.54±0.660	27.4±0.961	20.1±1.19	495±39.7	716±83.7	1.46±0.218
P value	0.37	0.88	0.001***	0.004**	0.64	<0.001***	0.003**

Data are reported as means±SEM. **P<0.01, ***P<0.001 (Mann-Whitney U test with Bonferroni correction). RF: resting flow; BZ: biological zero; PF: peak flow; AO: area of occlusion; AH: area of hyperemia; PU: perfusion units.

### Laboratory assay results

Supplementary Table S1 shows the serum test results from both groups at the end of the 23rd week. According to our findings, the HFD group had higher levels of leptin, MPO, CRP, OXLDL, LDL, OXLDL/LDL, TCHOL, TG, glucose, and HbA1c, but there was no statistical difference in VEGF and HDL levels between the groups.

### Correlation test results

**Figure 3:**
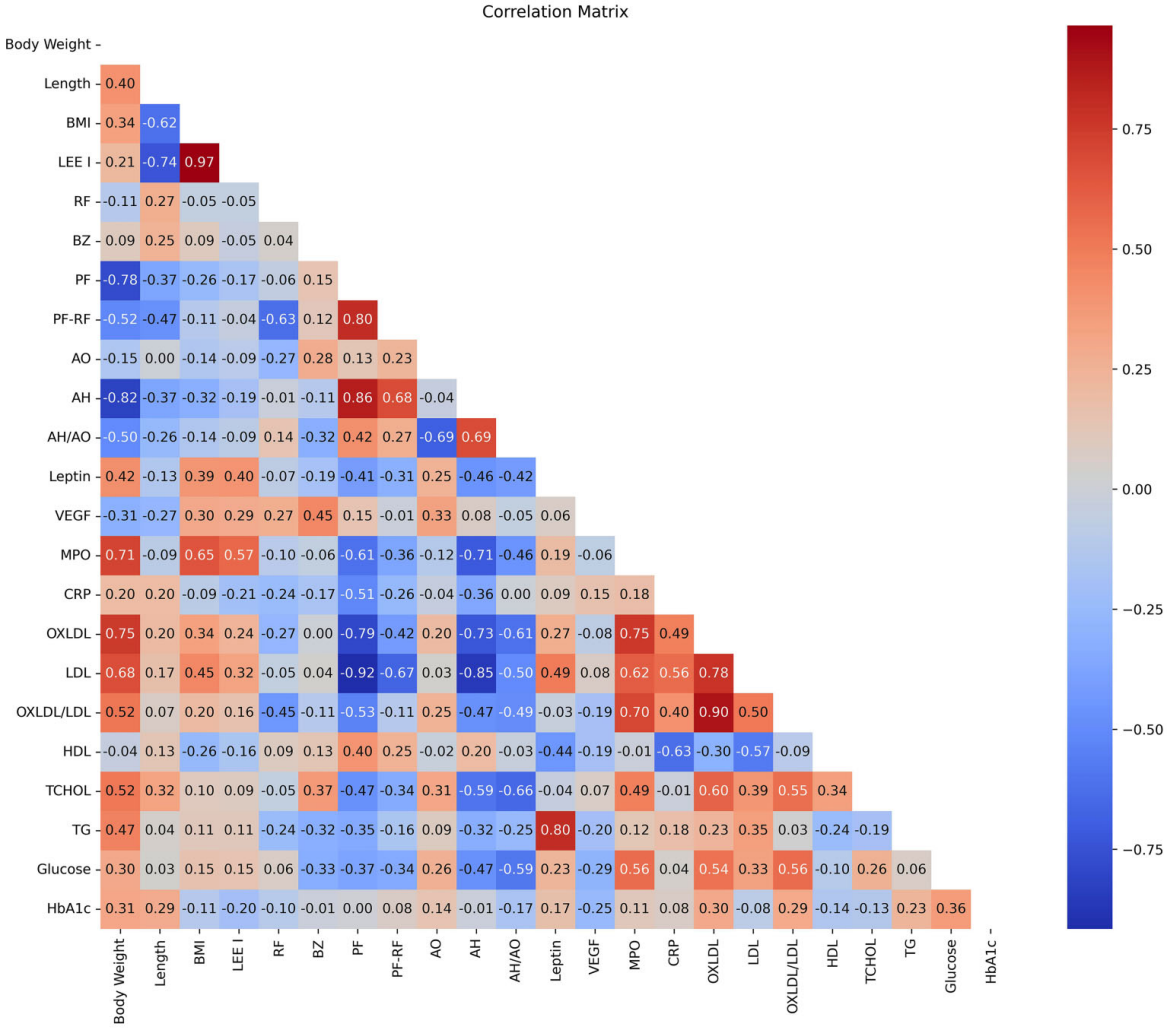
Correlation matrix table showing the relationship between parameters obtained from serum analysis, anthropometric measurements, and post-occlusion reactive hyperemia parameters at the end of the 23rd week in rats fed a high-fat diet. Relationships were classified as very strong (r≥0.8), strong (0.8>r≥0.6), moderate (0.6>r≥0.4), weak (0.4>r≥0.2), or negligible (r<0.2). BMI: body mass index; RF: resting flow; PF: peak flow; AH: area of hyperemia; VEGF: vascular endothelial growth factor; MPO: myeloperoxidase; CRP: C-reactive protein; OXLDL: oxidized low-density lipoprotein; LDL: low-density lipoprotein; HDL: high-density lipoprotein; TCHOL: total cholesterol; TG: triglycerides, HbA1c: hemoglobin-A1c.

In order to determine the relationship between the parameters obtained from serum analyses, anthropometric measurements, and PORH analyses in rats fed the high-calorie diet, we conducted a correlation test at the end of the 23rd week. Although the correlation test we conducted does not provide direct evidence of causality, it can inform us about causality and assist in our interpretation by determining the existence, direction, and strength of the relationship between the two variables (Figure 3).

#### Correlation between obesity and microvascular functions

Body weight showed a very strong negative correlation with AH and a strong negative correlation with PF. Body weight had a moderate negative correlation with PF-RF and AH/AO. The relationship between BMI and microvascular function markers was very weak. The Lee index did not show any correlation with these parameters.

#### Correlation between obesity and metabolic profiles

Body weight showed a strong correlation with LDL, a moderate correlation with TG and TCHOL, and a weak correlation with glucose and HbA1c, but no correlation with HDL. BMI and the Lee index showed a weaker or no correlation with the lipid profile parameters that body weight correlated with.

#### Correlation between metabolic profiles and microvascular functions

Microvascular function markers showed a very strong negative correlation with LDL, a moderate negative correlation with TCHOL, a weak negative correlation with TG, a moderate-weak negative correlation with glucose, a weak positive correlation with HDL, and no correlation with HbA1c.

#### Correlations of OXLDL

OXLDL had a strong correlation with body weight, however, its correlation with BMI and the Lee index was weak. The OXLDL/LDL ratio had a moderate correlation with body weight, but no correlation with BMI and the Lee index. LDL showed a strong correlation with OXLDL and a moderate correlation with OXLDL/LDL. Microvascular function markers had a strong negative correlation with OXLDL and a moderate negative correlation with the OXLDL/LDL ratio.

#### Correlations of leptin

Leptin had a moderate correlation with body weight and showed a similar correlation with BMI and the Lee index. Leptin had a very strong correlation with TG, a moderate correlation with LDL, a weak correlation with OXLDL, and no correlation with OXLDL/LDL. Leptin showed a moderate negative correlation with microvascular function parameters.

#### Correlations of MPO

MPO had a strong correlation with body weight and showed a similar correlation with BMI and the Lee index. MPO had a strong correlation with OXLDL, LDL, and OXLDL/LDL. MPO showed a strong negative correlation with PF and AH, a moderate negative correlation with AH/AO, and a weak negative correlation with PF-RF among microvascular function markers. MPO had a moderate correlation with TCHOL and glucose.

#### Correlations of VEGF

The correlation between VEGF and body weight, BMI, and the Lee index was negligible. VEGF showed no correlation with any metabolic profile parameters or microvascular function parameters. VEGF also did not correlate with OXLDL or the OXLDL/LDL ratio.

#### Correlations of CRP

The correlation between CRP and body weight, BMI, and the Lee index was negligible. CRP had a moderate correlation with OXLDL, LDL, and OXLDL/LDL, while showing a strong negative correlation with HDL. CRP showed a moderate negative correlation with PF and a weak negative correlation with AH among microvascular function markers, but no correlation with PF-RF and AH/AO.

Leptin, MPO, VEGF, and CRP showed no correlation with each other.

### In summary

According to these data, it can be said that vascular functions have a clear correlation only with body weight. Body weight had the most significant impact on microvascular functions, while BMI and the Lee index did not adequately reflect this relationship. It was observed that feeding a HFD primarily affected the lipid profile, especially LDL. The increase in OXLDL was seen together with the increase in LDL. Just as with the increase in LDL, the increase in OXLDL was also associated with a decrease in microvascular functions.

It is interesting that while leptin, MPO, and CRP increased in obesity, no relationship was observed between these parameters. Among these parameters, MPO had the strongest negative relationship with vascular functions. MPO also showed a strong relationship with OXLDL. When these results are evaluated together, it suggests that MPO, LDL, and OXLDL may be important in the deterioration of vascular functions and may have roles in this deterioration.

Although it is not correct to establish a causality relationship from the results of correlation tests, from our literature knowledge and the results we obtained, the following hypothesis can be generated: LDL increases due to obesity and OXLDL increases due to the rise in LDL and its increased oxidation. The activity of MPO or another stress source caused by obesity may lead to an increase in this oxidation. This oxidative condition itself or the OXLDL formed as a result of LDL oxidation may impair microvascular functions.

The strong negative correlation between CRP, an indicator of increased inflammation in obesity, and HDL suggests that elevated inflammation levels may reduce HDL levels, which in turn may diminish the cardiovascular protective effects of HDL. The partial correlation of both CRP and HDL with microvascular functions indicated that microvascular functions may be influenced not only by oxidative stress but also by inflammatory processes.

## Discussion

In this study, the complex and multifaceted relationships between microvascular functions and certain biomarkers were investigated in an obesity model. The results, while supporting some aspects of the literature, also present new information. The increase in leptin, MPO, CRP, LDL, OXLDL, TCHOL, TG, HbA1c, and glucose levels in obese rats, along with the impairment of microvascular functions, was an expected outcome based on the literature. The most significant biomarkers associated with the impairment of microvascular functions were LDL, OXLDL, MPO, and CRP. On the other hand, VEGF and the hormone leptin were not found to be associated with the impairment of microvascular functions.

While CRP functions as a biomarker of inflammation, MPO is associated with the production of free radicals during inflammatory processes (20). Both inflammation and oxidative stress increase with HFD. The increased oxidative stress due to HFD promotes the conversion of LDL to OXLDL (35). OXLDL is a potentially harmful type of LDL cholesterol responsible for endothelial dysfunction, atherosclerosis, and hypertension (36). The impairment of microvascular functions in obesity may not be solely due to LDL, but possibly due to the combination of LDL and oxidative stress. The increase in LDL, along with the rise in oxidant agents in the environment, increases the conversion of LDL to OXLDL, leading to a rise in the OXLDL/LDL ratio. Consequently, the relative increase in OXLDL will have negative effects on the vessels, impairing vascular functions. The positive correlation between HDL and vascular functions observed in the study also supports this hypothesis. This is because HDL reduces the adhesion and accumulation of OXLDL on the vascular walls (37). HDL is known to have protective effects against coronary heart disease. This effect may be due to its role in mitigating the effects of OXLDL. Perhaps, in addition to drugs used to reduce LDL cholesterol, developing drugs that prevent the conversion of LDL to OXLDL could be a new approach to preventing vascular diseases.

In a study conducted by Heslop et al. (20), it was found that assessing both MPO and CRP, rather than CRP alone, offered an enhanced ability to predict the risk of CAD. Because PORH and agents such as MPO and CRP are predictors of CAD, OXLDL, which is associated with all three, could potentially be a stronger predictor of CAD. There are several studies in the literature that support the likelihood of our hypothesis being correct (38). However, for a clearer understanding of this matter, more comprehensive research is required.

In our study, we observed a significant increase in serum leptin levels in rats with obesity models. This finding aligns with leptin's well-established role as a biomarker of fat mass. However, we did not detect a statistically significant increase in VEGF levels compared to the control group. The literature includes studies reporting either elevated VEGF levels or no change in obesity. In studies showing VEGF elevation, this phenomenon has often been linked to leptin, suggesting that leptin may upregulate VEGF synthesis to enhance angiogenesis and microvascular function (16-19). Conversely, there are also studies consistent with our results that found no association between obesity and VEGF or leptin-VEGF interactions (39,40). The potential reasons for these discrepancies among studies include: 1) Leptin resistance: the development of leptin resistance, a hallmark of obesity, may reduce signaling efficacy in target tissues despite hyperleptinemia, due to receptor desensitization or disruptions in cellular signaling pathways (6); 2) Dynamic nature of VEGF: VEGF levels may transiently rise under acute stress conditions such as inflammatory states or hypoxia (17,28,29). However, since obesity is a chronic condition, VEGF production might equilibrate over the long term; 3) Differences in study populations: variables such as age, sex, species (e.g., rats *vs* humans), or experimental models (e.g., cell cultures) could influence outcomes. Some studies involve young individuals, others older adults, or different gender groups; 4) Comorbid conditions: obesity-associated comorbidities, such as diabetes, might indirectly affect VEGF levels (15); 5) VEGF ısoform specificity: Discrepancies may stem from variability in assay specificity for VEGF isoforms (13). Many commercial kits lack explicit clarification of the targeted subtype (e.g., our “Rat VEGF ELISA Kit” was generically labeled, though its datasheet indicated higher specificity for VEGF-A). Inconsistent detection of isoforms across studies could confound total VEGF comparisons.

To resolve these contradictions, future studies should involve multicenter, long-term research designs with homogeneous groups that rigorously evaluate obesity stages, leptin signaling dynamics, and environmental factors.

In our study, we identified a significant correlation between leptin levels and TG and LDL cholesterol levels. Elevated TG and LDL are known to impair microvascular structure and function through processes such as endothelial dysfunction, oxidative stress, and atherosclerosis (3). Thus, even though we did not observe a direct relationship between leptin and microvascular function, it is plausible that leptin's adverse effects on lipid profiles indirectly modulate microvascular function. To validate this hypothesis, future studies must critically investigate the molecular and clinical impacts of the leptin-lipid relationship on endothelial function and vascular homeostasis.

A brief note should also be made regarding BMI and the Lee index. In the study, both BMI and the Lee index (which aims to more accurately assess body composition) showed a weaker correlation with the measured parameters compared to body weight. Height affected the calculation of BMI and the Lee index more than body weight. Since changes in height in rats differ according to the type of diet, using only body weight in such studies may be a more accurate method for assessing obesity in rats.

## Conclusions

This manuscript addressed a critical issue in obesity research by exploring the link between serum biomarkers and microvascular function. While obesity is known to cause weight gain and metabolic changes, the specific mechanisms connecting these biomarkers to vascular health are not well understood. Our study revealed that obesity-induced increases in certain biomarkers, such as OXLDL and inflammatory proteins, correlated strongly with impaired microvascular function. This new information highlights the role of oxidative stress and inflammation in vascular complications associated with obesity. By identifying these relationships, our findings pave the way for future research aimed at developing targeted interventions.

## Supplementary Material

Click to view [pdf].
