# Supplementary material for: Identification of serum biomarkers associated with microvascular functions in a long-term high-fat diet-induced obesity rat model
Source: Braz J Med Biol Res. 2025 Aug 29;58:e14811. doi: 10.1590/1414-431X2025e14811 (PMC12396620; doi:10.1590/1414-431X2025e14811)
Supplement: Supplementary file 1 [file 1414-431X-bjmbr-58-e14811-suppl.pdf]

Table S1. Analysis of substances in the sera of rats in the control and high-fat diet (HFD) groups at the end of the 23rd week.

	Leptin (ng/mL)	VEGF (pg/mL)	MPO (ng/mL)	CRP (mg/dL)	OXLDL (mg/dL)	LDL (mg/dL)	OXLDL/LDL	HDL (mg/dL)	TCHOL (mg/dL)	TG (mg/dL)	Glucose (mg/dL)	HbA1c (%)
Control (n=9)	2.22 ± 0.291	122 ± 6.17	18.5 ± 4.29	0.212 ± 0.011	8.43 ± 1.12	32.8 ± 4.31	0.259 ± 0.036	48.0 ± 5.10	83.5 ± 5.95	44.6 ± 11.7	92.8 ± 5.92	4.32 ± 0.234
HFD (n=14)	8.87 ± 1.44	125 ± 6.96	35.6 ± 9.29	0.329 ± 0.026	22.6 ± 9.05	47.9 ± 6.73	0.462 ± 0.150	44.9 ± 7.35	109 ± 8.78	98.9 ± 8.57	113 ± 5.77	4.82 ± 0.446
P value	<0.001***	0.44	<0.001***	<0.001***	<0.001***	<0.001***	<0.001***	0.19	<0.001***	<0.001***	<0.001***	0.004**

Data are reported as means±SEM. **P<0.01, ***P<0.001 (Mann-Whitney U test with Bonferroni correction). VEGF: vascular endothelial growth factor; MPO: myeloperoxidase; CRP: C-reactive protein; OXLDL: oxidized low-density lipoprotein, LDL: low-density lipoprotein, HDL: high-density lipoprotein; TCHOL: total cholesterol; TG: triglycerides; HbA1c: hemoglobin-A1c.
